# Ayurveda in Contemporary Healthcare: A Narrative Overview of Select Therapeutic Interventions and Their Emerging Clinical and Mechanistic Perspectives

**DOI:** 10.7759/cureus.108081

**Published:** 2026-05-01

**Authors:** Roshni Anirudhan, Amit Nampalliwar, Amar S Kamble, Dhanya R., Dipali Parekh, K. Parameswaran Namboothiri

**Affiliations:** 1 Department of Bal Roga (Pediatrics), Government Ayurveda College, Thiruvananthapuram, IND; 2 Department of Roga Nidan and Vikriti Vigyana (Diagnostics and Pathology), Government Ayurved College and Hospital, Bilaspur, IND; 3 Department of Rachana Sharir (Anatomy), Manjushree Research Institute of Ayurvedic Sciences, Gandhinagar, IND; 4 Department of Dravyaguna Vigyana (Pharmacology), Sumandeep Ayurved Medical College and Hospital, Sumandeep Vidyapeeth Deemed to be University, Vadodara, IND; 5 Department of Rasashastra and Bhaishajya Kalpana (Iatrochemistry and Pharmaceutics), Institute of Teaching and Research in Ayurveda, Jamnagar, IND; 6 Department of Panchakarma (Detoxification and Rejuvenation Therapy), Amrita School of Ayurveda, Amrita Vishwa Vidyapeetham, Kollam, IND

**Keywords:** ayurveda, botanical interventions, clinical integration, panchakarma, rasayana therapy

## Abstract

Ayurveda represents a long-standing medical tradition grounded in interconnected physiological, psychological, and environmental principles. Increasing interest in holistic and multi-targeted interventions has led to growing exploration of their therapeutic relevance in metabolic imbalance, chronic inflammation, neurocognitive decline, musculoskeletal dysfunction, and stress-associated disorders. Variations in study design, formulation quality, and mechanistic clarity continue to present challenges, and current evidence remains heterogeneous and often derived from small-scale or preliminary investigations, yet emerging scientific evaluations indicate preliminary clinical potential across several domains. This narrative review aimed to integrate selected Ayurvedic concepts with contemporary biomedical perspectives, based on a structured but non-systematic literature exploration rather than a formal systematic review methodology. The methodology involved a targeted, non-systematic search of publications from 2000 to 2025 across selected databases (e.g., PubMed, Scopus, and Google Scholar) to identify clinically relevant studies, mechanistic reports, and available safety-related observations focused on defined Ayurvedic interventions; however, no formal study selection criteria, quantitative synthesis, or bias assessment framework was applied. Findings from included reports suggest that botanical agents, *Rasayana *(rejuvenative therapy) formulations, *Panchakarma *(detoxification techniques) procedures, dietary measures, and mind-body practices have been associated with changes in metabolic indicators, inflammatory mediators, oxidative stress markers, autonomic responses, cognitive performance, and gastrointestinal function in specific study settings, although effect sizes, study designs, and reproducibility vary considerably. Across multiple clinical contexts, these interventions may support physiological resilience and functional outcomes as adjunctive approaches, but evidence remains context-dependent and not uniformly generalisable. Broader integration into modern healthcare settings will require improved standardisation, rigorous safety and toxicological evaluation, and methodologically robust clinical trials. Collectively, the available literature positions Ayurveda as a potential complementary approach warranting further critical investigation rather than definitive clinical adoption.

## Introduction and background

Ayurveda, derived from the Sanskrit words ‘*ayus*’ (life or longevity) and ‘*veda*’ (knowledge), is commonly translated as “knowledge of life,” and represents one of the oldest systems of traditional medicine, encompassing interconnected physiological, psychological, and environmental dimensions [1]. Its foundational constructs, including *dosha *(functional regulatory principles), *agni *(metabolic and digestive capacity), *dhatu *(tissue systems), *mala *(waste products), and *srotas *(biological channels of transport), are rooted in classical texts such as the Charaka Samhita and Sushruta Samhita, where health is defined as a state of equilibrium among these elements [2]. The* Tridosha *(three fundamental physiological principles) framework - *Vata*, *Pitta*, and *Kapha - *represents functional principles governing movement, transformation, and structural stability, respectively, and operates through specific qualities (*guna*), sites (*sthana*), and physiological actions (*karma*) that regulate bodily and mental processes. *Vata *is associated with kinetic activity and regulatory signalling, *Pitta *with metabolic transformation and biochemical processes, and *Kapha *with structural stability and lubrication; however, these roles are defined within a qualitative and systemic paradigm rather than discrete anatomical entities [3].

*Agni*, described as the central determinant of digestion and metabolism, extends beyond gastrointestinal function to include cellular transformation and nutrient assimilation at multiple levels (*jatharagni *(central digestive fire)* *and *dhatvagni* (tissue-level metabolic fire)), while *dhatu *refers to the sequentially formed tissue systems that sustain structural and functional integrity. The concept of *srotas *denotes dynamic channels responsible for transport and communication within the organism, linking intake, transformation, and elimination processes [4]. Ayurveda is traditionally understood within a broader holistic framework that includes related disciplines such as yoga, collectively emphasising balance between body, mind, and environment as central to health and well-being. *Rasayana*, a key therapeutic domain in Ayurveda, refers to rejuvenative interventions aimed at promoting longevity, tissue regeneration, and systemic resilience.

In contemporary interpretive models, these classical constructs have been tentatively correlated with neuroendocrine regulation, metabolic pathways, and tissue homeostasis; however, such mappings represent approximations and do not fully capture the integrative and qualitative nature of Ayurvedic theory. The relationship between *dosha*, *agni*, and *dhatu *is inherently interdependent, where imbalance (*vikriti*) arises from disruption in these functional networks, influenced by diet, lifestyle, environment, and psychological state [5]. While modern biomedical frameworks attempt to contextualise these principles through measurable physiological parameters, the original Ayurvedic model remains system-oriented, emphasising individualised constitution (*prakriti*), dynamic balance, and functional adaptability rather than reductionist mechanistic explanations. These concepts have gained renewed scientific attention with the emergence of molecular biology, neuroendocrine science, and integrative physiology, which offer potential but still evolving and partial biological correlates [3].

Interest in Ayurvedic interventions has expanded due to the global shift toward multidimensional strategies for chronic disease management [4]. The rising burden of metabolic disorders, inflammatory conditions, musculoskeletal degeneration, stress-related dysfunction, and lifestyle-associated morbidity has prompted exploration of complementary modalities beyond conventional pharmacotherapy [5]. Ayurveda encompasses botanical formulations, procedural interventions, dietary regulation, and mind-body practices aimed at modulating systemic function; however, the extent to which these interventions produce consistent and reproducible clinical outcomes remains variable across studies [6]. While compatibility with contemporary clinical pathways has been proposed, the supporting evidence base is uneven and context-dependent [7].

Significant barriers continue to limit mainstream clinical integration. Existing studies demonstrate substantial heterogeneity in design, sample size, intervention standardisation, and reporting quality, with a proportion of trials lacking reproducibility or yielding inconclusive outcomes, although this variability has not been systematically quantified in the current literature [8]. Variability in plant sourcing, cultivation conditions, and post-harvest processing complicates chemical standardisation of botanical preparations, thereby affecting reproducibility and dose predictability [9]. Limited pharmacokinetic and pharmacodynamic characterisation of commonly used herbs restricts accurate assessment of therapeutic range, bioavailability, and interaction risk [10]. Traditional procedural interventions such as *Panchakarma* remain insufficiently characterised in terms of biological mechanisms, dose-response relationships, and standardised clinical protocols [6]. *Panchakarma *refers to a group of structured therapeutic procedures involving detoxification and elimination techniques designed to restore physiological balance. Concerns regarding contamination, improper manufacturing practices, heavy metal exposure, and herb-drug interactions further underscore the need for strengthened quality control and regulatory oversight [8].

Despite these limitations, some controlled human studies report domain-specific effects of selected Ayurvedic interventions. *Withania somnifera*, *Curcuma longa*, *Tinospora cordifolia*, and *Commiphora mukul* have demonstrated anti-inflammatory, metabolic, neuroprotective, and immunomodulatory effects under defined study conditions, although effect sizes, population characteristics, and methodological rigour vary across trials [11,12]. Procedural interventions described as detoxification-oriented have shown preliminary effects in selected metabolic and inflammatory conditions, but lack standardised protocols and reproducible validation across independent studies [13]. Ayurvedic mind-body practices have been associated with measurable changes in autonomic regulation, stress biomarkers, sleep parameters, and psychological outcomes in specific cohorts, though generalisability remains limited [14]. These findings indicate investigational relevance rather than definitive therapeutic validation.

Contemporary scientific frameworks provide tools for interpreting Ayurvedic principles within quantifiable physiological parameters [3]. Advances in systems biology, metabolic science, neurobehavioral medicine, and oxidative stress research allow traditional constructs to be examined through measurable biomedical indicators, although such translational mappings remain partial and model-dependent. Omics-based approaches, including genomics, proteomics, and metabolomics, have been explored as tools to investigate Ayurvedic concepts such as *prakriti* and systemic regulation, although these applications remain exploratory and require further validation. Concepts such as *dosha *balance, *agni *regulation, *dhatu *nourishment, and mind-body integration have been explored in relation to neuroinflammation, oxidative stress, metabolic dysregulation, and cognitive function, but require more rigorous mechanistic validation [15]. These approaches enable outcome assessment using biomedical measures such as glycemic indices, lipid profiles, inflammatory markers, immune parameters, validated symptom scales, and cognitive performance metrics, though cross-study comparability remains limited [16].

A structured and critically balanced synthesis of traditional knowledge and contemporary evidence is necessary for rational clinical interpretation. Therapeutic efficacy, safety, formulation quality, and reproducibility require systematic and methodologically robust evaluation before integration into healthcare systems [17]. Such assessment facilitates identification of clinically relevant applications, limitations, and research gaps, while reducing the risk of overgeneralisation. Sustained public interest in holistic and preventive approaches underscores the importance of rigorous evaluation frameworks rather than descriptive expansion alone [18]. However, existing literature remains fragmented, with limited integration of classical Ayurvedic principles and contemporary clinical evidence within a unified framework, and insufficient emphasis on systematic mapping between traditional concepts and measurable biomedical outcomes. Ayurveda encompasses a wide range of interventions that may contribute to chronic disease management; however, their role should be interpreted within the context of evolving evidence, methodological limitations, the need for standardisation, and the current lack of integrative principle-to-evidence frameworks.

Objectives of the review

This narrative review aimed to examine selected Ayurvedic interventions by linking key principles (*dosha*, *agni*, *dhatu*) with reported biomedical outcomes, based on clinical and mechanistic studies primarily in adult populations.

The objectives were to: (i) map interventions (botanical, *Panchakarma*, dietary, mind-body) to underlying principles; (ii) summarise clinical outcomes across metabolic, inflammatory, neurocognitive, and stress-related domains; (iii) assess safety and reproducibility; and (iv) outline a preliminary translational perspective without applying formal systematic or omics-based models.

The scope was illustrative rather than comprehensive and did not establish clinical guidelines.

Methodology

For this narrative review, a structured, non-systematic literature search was conducted in PubMed, Scopus, Web of Science, and Google Scholar to identify relevant studies on Ayurvedic interventions published from 2000 to 2025 (search updated to present at the time of analysis). The core search string applied across databases (with minor adaptations) was: ("Ayurveda" OR "Ayurvedic medicine") AND ("herbal formulations" OR "botanical therapy" OR "*Panchakarma*" OR "mind-body practices") AND ("clinical outcomes" OR "metabolic" OR "inflammation" OR "cognitive" OR "safety").

The initial search yielded approximately 312 records across databases. After removal of duplicates (~72 records), 240 titles and abstracts were screened for relevance by a single reviewer based on predefined criteria aligned with the objectives of the review. Of these, 118 articles were excluded due to a lack of primary data, unclear intervention descriptions, or non-relevance to defined therapeutic domains. A total of 122 full-text articles were assessed, of which 48 studies were included in the final synthesis. Excluded studies reviewed at the screening stage generally demonstrated heterogeneous, inconclusive, or insufficiently detailed findings, including unclear intervention standardisation or inconsistent outcomes, and did not substantially contradict the overall interpretations derived from the included evidence.

Inclusion criteria comprised studies reporting clinical outcomes, mechanistic observations, or safety-related findings involving clearly defined Ayurvedic interventions (botanical, procedural, dietary, or mind-body). Mechanistic insights were operationally defined as studies reporting biochemical, physiological, or molecular effects linked to intervention exposure. Exclusion criteria included narrative opinions, anecdotal reports, studies lacking intervention clarity, and publications prior to 2000. Only English-language publications were considered; grey literature and unpublished data were not included, introducing potential publication bias.

No formal systematic review protocol (e.g., Preferred Reporting Items for Systematic Reviews and Meta-Analyses (PRISMA) or PRISMA Extension for Scoping Review (PRISMA-ScR)) was followed, and no standardised risk-of-bias or quality assessment tools (e.g., A Measurement Tool to Assess Systematic Reviews (AMSTAR), Grading of Recommendations Assessment, Development, and Evaluation (GRADE)) were applied. Study selection was based on relevance and conceptual alignment rather than methodological scoring, which may increase the risk of selection bias. This approach may have favoured the inclusion of studies reporting positive or interpretable outcomes over neutral or negative findings. A PRISMA-style flow structure is described narratively (identification: 312; screening: 240; eligibility: 122; included: 48), although a formal diagram was not constructed. As a non-systematic narrative review, this approach may not capture all relevant high-level evidence, including large randomised controlled trials, meta-analyses, or systematic reviews, and therefore should not be considered an exhaustive representation of the evidence base.

No quantitative synthesis (e.g., meta-analysis, pooled effect sizes, confidence intervals, or statistical comparisons) was performed due to heterogeneity in study design, interventions, and outcome reporting. Accordingly, this review should be interpreted as a thematic narrative synthesis rather than a reproducible systematic evidence appraisal, particularly given the single-reviewer screening process and absence of formal quality assessment.

Given the substantial heterogeneity in Ayurvedic interventions, including variability in formulations, procedural therapies, individualised treatment approaches, and outcome measures across clinical and mechanistic studies, a narrative (non-systematic) review design was considered more appropriate than a formal systematic review. The available evidence spans diverse study designs, ranging from exploratory mechanistic reports to small-scale clinical investigations, which limits the feasibility of standardised comparisons and quantitative synthesis. Therefore, a narrative approach was adopted to enable integrative, concept-driven synthesis and contextual interpretation of findings across domains, rather than strict methodological aggregation.

This narrative approach incorporates interpretive selection and thematic synthesis based on conceptual relevance, which inherently limits full methodological reproducibility. The absence of dual-review screening, formal risk-of-bias assessment, and standardised evidence grading further constrains replicability and may introduce subjective selection bias. Accordingly, the methodology supports structured transparency but should not be interpreted as a fully reproducible systematic protocol.

## Review

Evidence for botanical interventions

In Ayurvedic medicine, botanical preparations constitute a major therapeutic category and are increasingly explored in clinical and translational research, although findings remain heterogeneous across study designs [18]. Phytochemical constituents in these botanicals have been associated with changes in inflammatory, metabolic, neurocognitive, and immune pathways in experimental and limited clinical settings [19]. For example, *Commiphora mukul *has been reported to influence cytokine activity and metabolic signalling; however, these findings are context-specific and lack consistent dose-response characterisation, limiting generalisability [20]. While standardised extracts may improve comparability, variability in plant sourcing, processing, and potential adulteration continues to affect reproducibility and safety [21].

Clinical studies report associations between selected botanicals and changes in glycemic indices, lipid parameters, inflammatory markers, and cognitive measures; however, most evidence is derived from small samples (often <50 participants), short-duration trials, and single-study evaluations per agent [16,22]. Extracts such as curcuminoids, withanolides, guggulsterones, and triterpenoids have been investigated; however, limitations including low bioavailability (notably for curcumin), inconsistent formulation standardisation, and limited pharmacokinetic data constrain the interpretation of outcomes [21]. Reported mechanisms, including antioxidant, anti-inflammatory, and metabolic regulatory effects, are frequently inferred from preclinical models and remain inconsistently validated in human studies, contributing to variability in translational relevance [15].

Within classical Ayurveda, botanical interventions are individualised based on *dosha *predominance and *agni *status, a principle that is not incorporated into most contemporary clinical study designs. This divergence reflects a broader methodological gap between traditional individualised therapeutic frameworks and standardised clinical trial models, which may partially explain inconsistencies in observed outcomes and limit direct cross-study comparability.

Advances in extraction and phytochemical characterisation have improved formulation consistency; however, challenges related to contamination, adulteration, and regulatory variability persist [9]. As reflected in Table 1, many botanicals are supported by single-study evidence without standardised dosing or replicated validation, limiting the strength and reproducibility of conclusions. 

**Table 1:** Key Ayurvedic botanicals, therapeutic domains, and evidence overview CRP: C-reactive protein; NK cell: Natural killer cell; LDL-C: Low-density lipoprotein cholesterol; HDL-C: High-density lipoprotein cholesterol

Botanical Agent	Active Constituents	Therapeutic Domain	Key Clinical Outcomes	Dose / Formulation	Sample Size (n)	Study Type	Population	Effect Reporting	*Dosha */ *Prakriti *Context	Reference
Withania somnifera	Withanolides	Stress modulation, cognition	Reduced cortisol, improved attention scores	Standardised extract (300-600 mg/day; varies by study)	Typically <60 participants	Randomised controlled trial (small-scale)	Adults with stress or mild cognitive impairment	Statistical significance reported; effect sizes inconsistently defined	Traditionally indicated for *Vata *imbalance (stress, fatigue, neuromuscular weakness)	Alanazi and Elfaki, 2023 [12]
Curcuma longa	Curcuminoids	Inflammation, metabolism	Lower CRP, improved glycemic indices	Curcumin extracts (500-1500 mg/day; bioavailability-dependent formulations)	Generally <100 participants	Randomised or controlled clinical studies	Adults with metabolic or inflammatory conditions	Variable reporting; limited standardised effect size metrics	Associated with *Kapha-Pitta *imbalance (inflammation, metabolism)	Uchio et al., 2024 [21]
Tinospora cordifolia	Alkaloids, glycosides	Immune regulation	Enhanced NK cell activity, reduced fatigue scores	Extract doses ~300-500 mg/day equivalent	Typically <80 participants	Prospective or controlled clinical studies	Adults with immune dysfunction or fatigue-related conditions	Outcome-based reporting; limited quantitative synthesis	Traditionally used in *Pitta *imbalance and immune dysregulation	Gupta et al., 2024 [11]
Commiphora mukul	Guggulsterones	Lipid regulation	Decreased LDL-C, improved HDL-C ratio	Standardised guggul extract (500-1000 mg/day)	Typically <70 participants	Controlled clinical trials	Adults with dyslipidemia	Inconsistent reporting of confidence intervals and effect magnitude	Traditionally applied in *Kapha *disorders (lipid metabolism)	Garang et al., 2023 [20]
Centella asiatica	Triterpenoids	Neurocognitive support	Improved memory scores, reduced oxidative markers	Extract doses ~300-600 mg/day equivalent	Typically <50 participants	Small randomised or pilot studies	Adults with cognitive decline or healthy volunteers	Descriptive outcomes; limited effect size reporting	Associated with *Vata*-*Pitta *balance (cognitive and neural function)	Gray et al., 2018 [9]

*Rasayana *therapy

*Rasayana, *a rejuvenative therapeutic approach in Ayurveda, is traditionally described as supporting vitality, tissue integrity, and long-term functional capacity, rather than uniformly enhancing systemic resilience across all contexts [23]. Classical descriptions associate *Rasayana *with nourishment (*poshana*), cognitive clarity, and sustained physiological function; however, these effects are conceptual, individualised, and not directly comparable across populations [23]. Contemporary studies have explored associations with antioxidant activity, immunomodulation, and metabolic regulation during ageing, but findings remain variable and inconsistent, including reduced or non-uniform effects in elderly cohorts [24]. These observations suggest possible relevance in preventive and supportive contexts rather than definitive clinical efficacy [25].

Commonly studied *Rasayana *agents include *Emblica officinalis*, *Withania somnifera*, *Tinospora cordifolia*, and *Glycyrrhiza glabra*, although these represent only a limited subset of a broader classical pharmacopoeia [26]. These botanicals have been associated with modulation of oxidative stress, inflammatory pathways, and metabolic processes in experimental and small clinical studies; however, reported effects are context-dependent and not consistently supported by large-scale or comparative trials [27]. Proposed adaptogenic effects, including neuroendocrine and autonomic modulation, remain largely inferential and lack consistent validation in human populations [28,29].

Reported outcomes such as changes in antioxidant markers, lipid profiles, cognitive measures, and fatigue indices vary in magnitude and are typically derived from small, heterogeneous studies with limited standardisation and follow-up [30]. These outcomes show considerable variability across study populations and intervention protocols, limiting reproducibility and cross-study comparability. Evidence suggesting regenerative effects, including wound healing and tissue recovery, is restricted to specific study settings and requires replication in larger controlled trials [31]. These findings are primarily associative and do not establish causal mechanisms.

Within classical Ayurvedic practice, *Rasayana *is applied as part of an integrated therapeutic strategy involving dietary regulation, detoxification procedures, and behavioural interventions aimed at optimising *agni *and supporting *dhatu *integrity. This integrative and individualised application is not reflected in most contemporary clinical studies, where isolated agents are evaluated under standardised conditions, contributing to discrepancies between traditional practice and reported outcomes.

**Figure 1:**
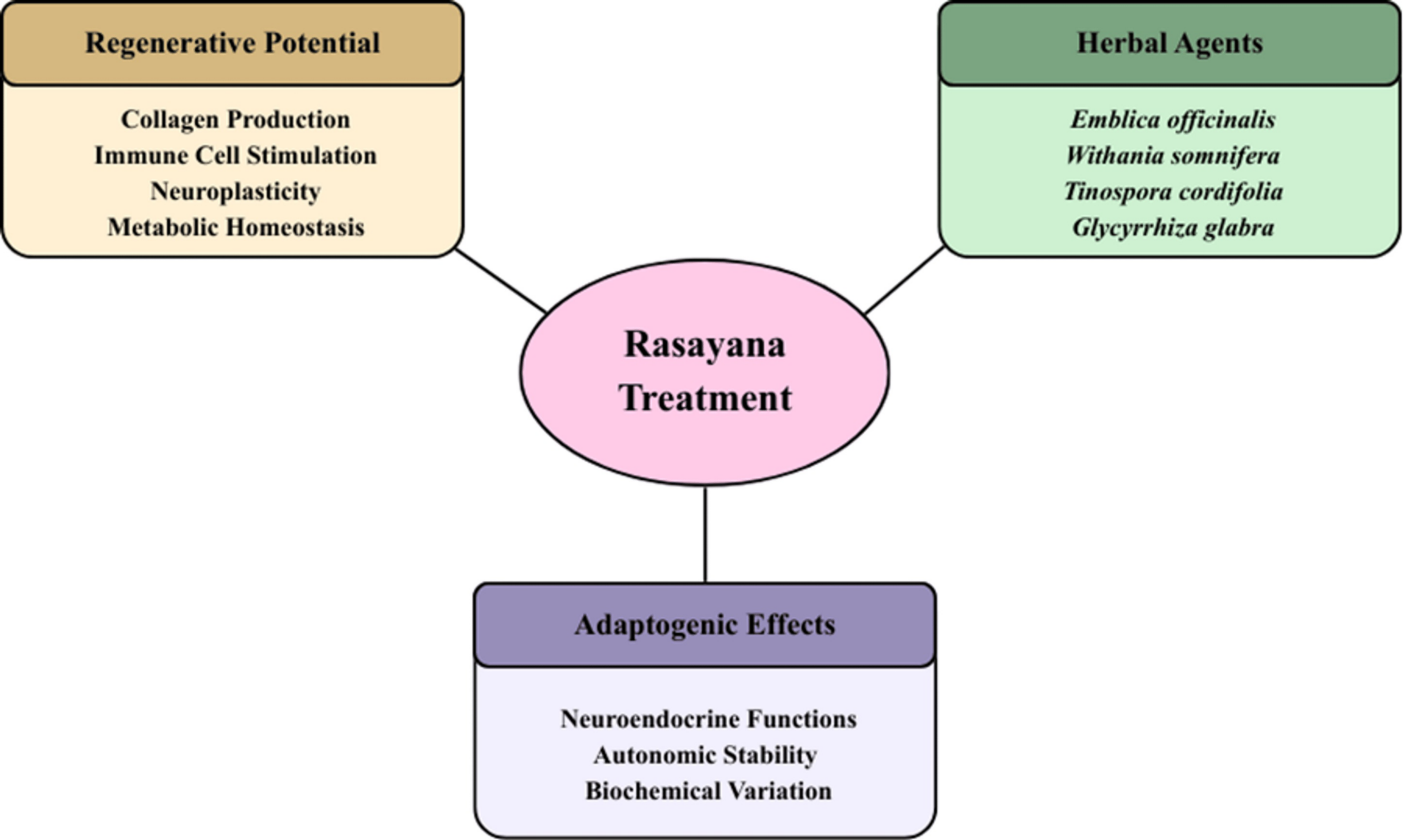
Overview of Rasayana treatment mechanisms Image created by authors using Canva (Canva Pty Ltd., Sydney, Australia).

Overall, the evidence base for *Rasayana *therapy remains fragmented, with variability in study design, population characteristics, and outcome reporting. The therapeutic scope extends beyond the limited number of commonly studied agents, and current literature does not fully represent this diversity. Figure 1 illustrates proposed mechanisms and representative agents; however, these should be interpreted as conceptual associations rather than definitive or causal relationships.

*Panchakarma *procedures

*Panchakarma, *a group of structured detoxification and eliminative techniques in Ayurveda, is a principal therapeutic system involving a sequence of preparatory and eliminative interventions intended to restore physiological balance, although its effects remain context-dependent and not consistently validated across clinical settings [6]. Classical descriptions outline five procedures - *Vamana *(therapeutic emesis), *Virechana *(therapeutic purgation), *Basti *(medicated enema therapy), *Nasya *(nasal administration therapy), and *Raktamokshana *(therapeutic bloodletting) - each directed toward specific functional pathways rather than discrete anatomical targets [1]. Contemporary studies have reported associations with changes in inflammatory markers, autonomic regulation, and metabolic parameters; however, these findings are derived largely from small-scale or low-quality studies with limited standardisation [24]. The preparatory phase, involving oleation and sudation, is considered essential for mobilising metabolites, but protocols related to duration, dosage, and frequency are inconsistently reported, limiting reproducibility [32].

Reported changes in oxidative stress markers, lipid profiles, glycemic indices, and cytokine patterns vary across study designs and lack consistent validation. Emerging observations related to gut microbiota, endocrine responses, and mitochondrial function remain exploratory and require confirmation in controlled trials [13]. Individual procedures have been studied in limited contexts: *Basti *has been associated with modulation of autonomic tone and gut-brain signalling, but safety considerations such as electrolyte imbalance and infection risk are not consistently evaluated [30]; *Virechana *and *Vamana *have been linked to changes in metabolic and hepatic parameters, though standardisation and patient selection remain unclear [33]; and *Nasya *has shown associations with upper airway function and selected neurocognitive outcomes, with heterogeneous evidence [15]. These findings remain procedure-specific and lack consistent replication across independent clinical settings, limiting generalisability.

Within classical practice, *Panchakarma *is applied as part of an integrated therapeutic approach alongside dietary regulation and botanical interventions to support *agni *and systemic balance. This integrative and individualised application contrasts with contemporary research models, where procedures are often evaluated in isolation, contributing to variability in reported outcomes and limiting alignment with traditional practice. Overall, current evidence remains preliminary, with heterogeneity in study design, limited standardisation, and inconsistent safety reporting. Table 2 summarises the principal *Panchakarma *procedures and associated observations, which should be interpreted cautiously given methodological limitations and the absence of large-scale or comparative clinical validation.

**Table 2:** Panchakarma procedures and associated clinical indicators GI: Gastrointestinal

Procedure	Target System	Primary Therapeutic Actions	Key Clinical Indicators	Typical Protocol (Variability Noted)	Study Type / Population	Safety Considerations	Reference
Vamana	Upper GI tract	Elimination of accumulated mucus	Reduced congestion, lowered inflammatory load	Single-day emesis procedure following 3-7 days of oleation/sudation (varies)	Small clinical studies; adults with respiratory/metabolic conditions	Risk of dehydration, electrolyte imbalance, procedural intolerance	Gupta, 2021 [30]
Virechana	Hepatic-intestinal axis	Removal of metabolic by-products	Improved lipid profile, hepatic markers	Purgation following preparatory oleation (3-7 days); dose varies by formulation	Controlled clinical observations; adults with metabolic disorders	Dehydration, electrolyte shifts, gastrointestinal discomfort	Gupta, 2021 [30]
Basti	Gut-brain axis	Modulation of autonomic and inflammatory pathways	Balanced autonomic tone, reduced cytokine levels	Series of medicated enemas over multiple days (commonly 8-30 sessions)	Experimental/clinical studies; adults with neurological or inflammatory conditions	Electrolyte imbalance, infection risk, procedural variability	Bonaz et al., 2018 [34]
Nasya	Upper respiratory-cranial interface	Nasal clearance, neural stimulation	Improved airflow, cognitive parameters	Daily or periodic nasal instillation (duration varies widely)	Small-scale studies; mixed adult populations	Irritation, improper administration risks	Leupin and Britz, 2025 [15]
Raktamokshana	Vascular system	Removal of impure blood fractions	Reduced localised inflammation, oxidative markers	Bloodletting using leeches or venesection; frequency variable	Limited clinical reports; localised inflammatory conditions	Infection risk, anaemia, procedural complications	Katrodiya et al., 2017 [24]

Management of metabolic and lifestyle disorders

Ayurvedic management of metabolic and lifestyle disorders involves botanical agents, dietary regulation, procedural interventions, and mind-body practices, although evidence remains heterogeneous and context-dependent [25]. Core principles include optimisation of digestive and metabolic function (*agni*), reduction of inflammatory load, and neuroendocrine stabilisation; however, *agni *lacks direct biomedical markers, limiting clinical quantification [35]. This limitation reflects a broader challenge in translating qualitative Ayurvedic constructs into measurable biomedical parameters, contributing to variability in clinical interpretation.

Botanicals such as *Curcuma longa*, *Gymnema sylvestre*, *Commiphora mukul*, *Emblica officinalis*, and *Tinospora cordifolia *have been associated with changes in glucose metabolism, lipid profiles, and inflammatory markers, but findings are inconsistent and largely derived from small or short-duration studies without replication [36,37]. Procedural interventions (*Virechana*, *Basti*) and mind-body practices show potential effects on metabolic and autonomic parameters, though mechanisms remain unclear and protocols are not standardised [6,14]. Reported effects across these intervention categories vary in magnitude and are not consistently reproducible across study populations, limiting generalisability.

**Figure 2:**
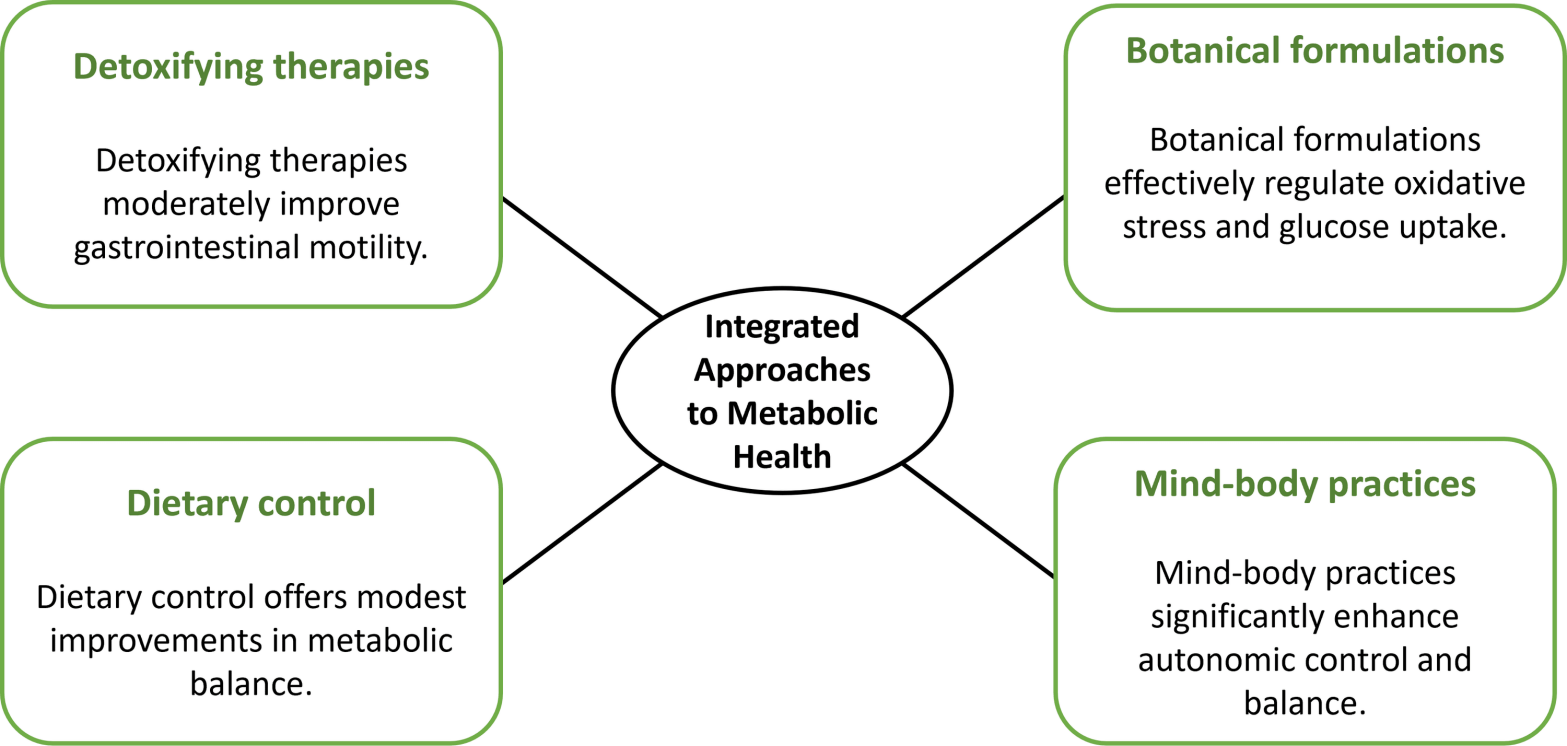
Key Ayurvedic approaches for managing metabolic disorders Image created by authors using Napkin AI (Second Layer, Inc., Los Altos, USA).

Potential herb-drug interactions, such as with *Gymnema sylvestre *and metformin, require clinical consideration. In classical practice, these interventions are combined to regulate *agni*; however, most studies evaluate them in isolation. This divergence between integrative traditional application and reductionist study designs may contribute to inconsistent outcomes and limit interpretation within a holistic Ayurvedic framework. Overall, evidence suggests possible adjunctive benefits in early metabolic dysfunction but remains limited by small sample sizes, variability, and lack of standardised reporting. Figure 2 should be interpreted as representing associative, not causal, relationships.

Ayurvedic strategies for pain and musculoskeletal conditions

Ayurvedic management of musculoskeletal disorders involves botanical agents, external therapies, *Panchakarma *procedures, and movement-based approaches aimed at reducing inflammation, improving joint function, and supporting neuromuscular coordination, although evidence remains variable and largely derived from small or non-comparative studies [38,39]. Core principles include balancing functional disturbances (e.g., *Vata*-related conditions), improving tissue lubrication, and enhancing circulation, but these are not consistently applied in clinical study designs [1]. Botanicals such as *Boswellia serrata*,* Curcuma longa*, *Tinospora cordifolia*, *Zingiber officinale*, and *Withania somnifera* have been associated with reductions in pain, stiffness, and functional limitation; however, most studies involve small samples, short durations, and, in some cases, potential funding bias, with limited comparison to standard treatments such as non-steroidal anti-inflammatory drugs (NSAIDs) [11,12,40,41].

External therapies (*Abhyanga *(therapeutic oil massage), *Pinda Sweda* (herbal bolus sudation therapy), *Kati Basti* (localised oil retention therapy over the lumbar region)) and *Panchakarma *procedures show reported benefits in pain and mobility, but protocols are highly variable and lack standardisation, limiting reproducibility [6]. Mind-body and movement-based interventions demonstrate effects on pain perception and autonomic regulation, though these are not specific to Ayurvedic frameworks [19]. Overall, evidence reflects a multi-component approach but remains limited by heterogeneity, small sample sizes, lack of standardised protocols, and minimal comparative trials, restricting definitive conclusions.

Neurocognitive and psychiatric applications

Ayurvedic management of neurocognitive and psychiatric conditions focuses on the regulation of stress responses, support of cognitive function, and stabilisation of neural processes, although evidence remains variable and largely derived from small studies [15]. Core principles include mind-body regulation and nourishment of cognitive function; however, these are not consistently operationalised in clinical research [42]. This reflects a broader limitation in translating integrative and qualitative Ayurvedic constructs into standardised and measurable clinical endpoints. Botanical agents classified as *Medhya Rasayanas* (cognitive-supportive Ayurvedic rejuvenative agents), including *Withania somnifera*, *Bacopa monnieri*,* Centella asiatica*, and *Glycyrrhiza glabra*, have been associated with changes in cognitive performance, stress markers, and sleep parameters [12,16,43]. Proposed mechanisms such as cholinergic modulation, antioxidant effects, and neuroinflammatory regulation remain largely inferential and lack consistent pharmacokinetic or causal validation. Reported improvements in memory, attention, and anxiety are derived from small, heterogeneous studies without standardised outcome reporting [14]. These findings show variability across study populations and intervention protocols, limiting reproducibility and generalisability.

Mind-body practices, including breathing and meditation, show effects on autonomic regulation, cortisol levels, and perceived stress; however, these effects are not specific to Ayurvedic frameworks and may be influenced by behavioural and contextual factors [14,19]. This overlap with broader psychophysiological interventions makes attribution of effects specifically to Ayurvedic principles methodologically challenging. *Panchakarma *interventions such as *Nasya *and *Basti *have been associated with neuroendocrine and gut-brain effects, but evidence remains preliminary and inconsistently reported [6]. These observations are largely exploratory and lack replication in well-controlled clinical settings. The findings suggest possible benefits in mild cognitive and stress-related conditions, but evidence is limited by small sample sizes, variability, and lack of standardised protocols. Evidence in severe psychiatric disorders remains limited.

Table 3 summarises key interventions and should be interpreted as reflecting associative, not causal, relationships.

**Table 3:** Key Ayurvedic agents for neurocognitive and psychiatric support HRV: Heart rate variability; PK: Pharmacokinetic; PD: Pharmacodynamic

Botanical Agent / Intervention	Active Components / Mechanism	Target Domain	Key Clinical Outcomes	Study Type / Population	Mechanistic Evidence Level	Reference
Bacopa monnieri	Bacosides	Memory, learning	Enhanced retention, improved processing speed	Small randomised controlled trials; adults with mild cognitive impairment or healthy volunteers	Mechanisms proposed (e.g., synaptic modulation), but limited PK/PD validation	McPhee et al., 2021 [16]
Withania somnifera	Withanolides	Stress, cognition	Lower cortisol, improved attention scores	Randomised or controlled trials; adults with stress-related conditions	Proposed neuroendocrine modulation; limited direct mechanistic confirmation	Alanazi and Elfaki, 2023 [12]
Centella asiatica	Triterpenoids	Neuroprotection	Reduced oxidative markers, improved recall	Small clinical studies; mixed adult populations	Associative antioxidant mechanisms; limited causal evidence	Gray et al., 2018 [9]
*Nasya *therapy	Nasal herbal oils	Limbic pathways	Improved sleep quality, emotional parameters	Observational or small clinical studies; limited standardisation	Mechanistic pathways speculative; lacks direct neurobiological validation	Acharya, 2024 [6]; Leupin and Britz, 2025 [15]
Meditative breathing	Autonomic modulation	Anxiety, stress	Improved HRV, reduced tension scores	Controlled and observational studies; diverse populations	Well-supported autonomic effects, but placebo and expectancy influences not consistently controlled	Khumalo, 2025 [14]; Leventhal et al., 2008 [19]

Dermatologic and gastrointestinal therapeutics

Ayurvedic approaches to dermatologic and gastrointestinal disorders focus on improving skin balance, supporting digestion, and reducing inflammation through herbal therapies, diet, and procedures, although the evidence base remains limited and methodologically inconsistent [1]. Traditional concepts link skin health to digestive function (*agni*), but this relationship has not been clearly validated in biomedical terms, and its alignment with modern scientific frameworks remains largely conceptual [2,34]. Herbal agents such as *Azadirachta indica*, *Curcuma longa*, *Hemidesmus indicus*, *Rubia cordifolia*, *Zingiber officinale*, *Piper longum*, *Glycyrrhiza glabra*, and *Tinospora cordifolia* have shown potential in improving symptoms like erythema, pruritus, and digestive discomfort; however, findings are largely derived from small or non-standardised studies with limited use of validated outcome measures such as Psoriasis Area and Severity Index (PASI) scores [30,44,45]. *Panchakarma *procedures, including *Virechana *and *Basti*, have been explored for their effects on gut function and microbial balance, but current evidence remains preliminary [46]. Variability in formulations, lack of protocol standardisation (particularly for external applications), and limited safety reporting, including potential hepatotoxicity and drug interaction, further constrain reproducibility and risk-benefit assessment [8]. Overall, more rigorous and standardised clinical studies are needed to clarify effectiveness and safety.

Safety, toxicology, and quality control

Safety remains a critical component of Ayurvedic therapeutics, as clinical utility depends on consistent quality, appropriate dosing, and toxicological evaluation [8]. Phytochemical variability due to plant sourcing, environmental conditions, and processing, along with risks of contamination (e.g., heavy metals, pesticides), complicates the prediction of efficacy and safety [36]. This variability introduces uncertainty in dose-response relationships and contributes to inconsistent clinical outcomes across studies. Strengthened quality control and standardisation are therefore essential to improve reproducibility and reduce adverse events [19].

Toxicological studies indicate dose-dependent effects on hepatic, renal, and gastrointestinal parameters, highlighting the need for regulated dosing and monitoring [26,47]. Standardised extracts with defined constituent profiles may reduce variability, though safety data remain limited. Pharmacovigilance systems and post-marketing surveillance are important for identifying rare adverse events and herb-drug interactions [10]. However, reporting of adverse events in existing studies is often inconsistent or incomplete, limiting comprehensive safety assessment.

Quality control frameworks increasingly incorporate raw material authentication, chromatographic profiling, and contaminant testing. Practices such as good manufacturing practice (GMP) and analytical techniques (e.g., high-performance liquid chromatography (HPLC), gas chromatography-mass spectrometry (GC-MS), DNA barcoding) support consistency and detection of adulteration [48]. Despite these methodological advances, implementation remains variable across regions and manufacturers, and regulatory harmonisation is limited. Continued emphasis on safety evaluation, standardisation, and regulatory oversight is required to ensure reliable clinical application.

Pathways for clinical integration

Integration of Ayurvedic therapies into clinical care requires alignment between traditional principles, contemporary evidence standards, and routine medical practice [1]. Effective implementation depends on clear therapeutic indications, standardised protocols, defined safety parameters, and measurable outcomes [17]. This alignment remains incomplete, as differences between individualised Ayurvedic approaches and standardised biomedical frameworks present ongoing methodological challenges. Current evidence suggests potential for combining botanicals, procedural interventions, dietary regulation, and mind-body practices as adjuncts in metabolic, inflammatory, neurocognitive, and stress-related conditions; however, findings remain context-dependent and not uniformly validated [5].

Objective evaluation using biochemical markers, symptom indices, and quality-of-life measures can support clinical assessment, though standardised application remains limited [6]. Variability in outcome selection and measurement further restricts cross-study comparability and clinical translation. Interprofessional collaboration and regulatory oversight, including quality control and pharmacovigilance, are essential for safe integration [8]. Digital tools may facilitate monitoring and documentation, but their role in Ayurvedic practice is still emerging [17]. Evidence supporting digital integration remains preliminary and requires validation in clinical settings. Overall, integration requires standardised methodologies, safety assurance, and interdisciplinary coordination, while current evidence supports cautious and selective application rather than routine clinical adoption.

Limitations and future recommendations

Current evidence in Ayurvedic therapeutics remains constrained by inconsistencies in study design, variability in formulation standardisation, and limited mechanistic characterisation. Small sample sizes and short intervention durations reduce generalisability to long-term outcomes, while cross-study comparisons remain challenging due to heterogeneity in botanical sourcing, processing methods, and diagnostic frameworks. In addition, insufficient toxicological data and underreporting of adverse events limit accurate safety assessment across diverse populations. Future research should prioritise standardisation of formulations, precise dose calibration, and structured clinical monitoring to enhance reproducibility, alongside strengthened pharmacovigilance systems, comprehensive toxicological evaluation, and authentication of raw materials to improve safety. Interdisciplinary collaboration among clinicians, pharmacologists, and biomedical scientists is required to elucidate mechanistic pathways, while the adoption of validated outcome measures, electronic surveillance systems, and molecular profiling techniques may improve therapeutic assessment across domains. Overall, Ayurvedic interventions are best interpreted as multi-component, context-dependent approaches with heterogeneous and evolving evidence, and the present conclusions reflect a narrative synthesis rather than quantitative pooled inference.

## Conclusions

This review outlines a range of Ayurvedic therapeutic interventions and their potential relevance in contemporary clinical contexts; however, the evidence base remains heterogeneous, largely derived from small-scale studies, and limited by variability in design, standardisation, and reporting. Reported effects on oxidative stress, inflammatory pathways, metabolic regulation, neurocognitive function, and tissue repair indicate possible physiological associations, but these findings are inconsistently validated and should be interpreted cautiously. The narrative design introduces potential selection bias and lacks formal quality appraisal, which may overrepresent favourable outcomes. Future research requires rigorously designed randomised controlled trials with standardised protocols, clearly defined clinical endpoints, and comprehensive safety evaluation, including pharmacokinetic profiling and herb-drug interaction assessment. Comparative effectiveness studies and the incorporation of individualised Ayurvedic frameworks may further clarify clinical applicability. Ayurveda may be considered a complementary approach in selected contexts; however, broader integration depends on the development of a robust, reproducible, and critically evaluated evidence base.
